# The Impact of Primary Tumor Location in Synchronous Metastatic Colorectal Cancer: Differences in Metastatic Sites and Survival

**DOI:** 10.1245/s10434-019-08100-5

**Published:** 2019-12-02

**Authors:** Nelleke P. M. Brouwer, Dave E. W. van der Kruijssen, Niek Hugen, Ignace H. J. T. de Hingh, Iris D. Nagtegaal, Rob H. A. Verhoeven, Miriam Koopman, Johannes H. W. de Wilt

**Affiliations:** 1 Department of Surgery, Radboud University Medical Center, Nijmegen, The Netherlands; 2 Department of Medical Oncology, University Medical Center Utrecht, Utrecht, The Netherlands; 3 Department of Surgery, Catharina Hospital Eindhoven, Eindhoven, The Netherlands; 4 Department of Pathology, Radboud University Medical Center, Nijmegen, The Netherlands; 5 Department of Research, Netherlands Comprehensive Cancer Organization (IKNL), Utrecht, The Netherlands

## Abstract

**Purpose:**

We explored differences in survival between primary tumor locations, hereby focusing on the role of metastatic sites in synchronous metastatic colorectal cancer (mCRC).

**Methods:**

Data for patients diagnosed with synchronous mCRC between 1989 and 2014 were retrieved from the Netherlands Cancer registry. Relative survival and relative excess risks (RER) were analyzed by primary tumor location (right colon (RCC), left colon (LCC), and rectum). Metastatic sites were reported per primary tumor location. Survival was analyzed for metastatic sites combined and for single metastatic sites.

**Results:**

In total, 36,297 patients were included in this study. Metastatic sites differed significantly between primary tumor locations, with liver-only metastases in 43%, 54%, and 52% of RCC, LCC, and rectal cancer patients respectively (*p* < 0.001). Peritoneal metastases were most prevalent in RCC patients (33%), and lung metastases were most prevalent in rectal cancer patients (28%). Regardless of the location of metastases, patients with RCC had a worse survival compared with LCC (RER 0.81, 95% CI 0.78–0.83) and rectal cancer (RER 0.73, 95% CI 0.71–0.76). The survival disadvantage for RCC remained present, even in cases with metastasectomy for liver-only disease (LCC: RER 0.66, 95% CI 0.57–0.76; rectal cancer: RER 0.84, 95% CI 0.66–1.06).

**Conclusions:**

This study showed significant differences in relative survival between primary tumor locations in synchronous mCRC, which can only be partially explained by distinct metastatic sites. Our findings support the concept that RCC, LCC and rectal cancer should be considered distinct entities in synchronous mCRC.

Colorectal cancer (CRC) is the third most common cancer in the world.^1^. Approximately 25% of the patients present with synchronous metastatic colorectal cancer (mCRC).^2^ In a time span of 25 years, the relative survival of both early-stage colorectal cancer and mCRC has improved. The 5-year relative survival of mCRC increased from 4 to 12%, mainly due to intensified and more effective treatment.^3^

Primary tumor location in CRC has prognostic value, suggesting that right-sided colon (RCC), left-sided colon (LCC), and rectal cancer can be regarded as different entities. Population-based studies found that patients with metastasized LCC had better survival compared with patients with metastasized RCC and demonstrated that in recent years patients with metastasized rectal cancer had a better survival than patients with metastasized colon cancer.^2^,^4^

There are several explanations for the differences in prognosis between primary tumor locations: (1) poorer response to systemic therapy of patients with RCC patients compared with patients with LCC; (2) higher frequency of signet-ring cell carcinoma and mucinous adenocarcinoma in RCC, which are associated with worse outcome; and (3) differences in metastatic patterns, which might influence survival as well.^5^–^10^

The purpose of this study was to explore the differences in survival between primary tumor location in mCRC, hereby focusing on the role of metastatic sites using data from the Netherlands Cancer Registry.

## Methods

### Patient and Data Collection

Data were retrieved from the Netherlands Cancer Registry (NCR), which collects data of all patients with newly diagnosed malignancies in the Netherlands since 1989. Trained registrars gather data on tumor and treatment characteristics, after notification from the pathology departments of hospitals, all taking part in the automated pathology archive (PALGA), and the National Registry of Hospital Discharge Diagnoses. Primary tumor characteristics were coded according to International Classification of Diseases for Oncology (ICD-O) and the TNM (tumor-node-metastasis) classification, using the edition valid at time of cancer diagnosis.^11^,^12^

All CRC patients who were diagnosed between 1989 and 2014 with distant metastases at the time of diagnosis (i.e., synchronous metastases) were selected. Synchronous metastases were defined as distant metastases of primary CRC in other organs, excluding regional lymph nodes, detected by imaging or histological techniques previous to the start of treatment. Anatomical sites of metastases were registered according to the ICD-O. Since 2008, the registration of metastases was fully implemented in all regions in the Netherlands. Patients with unknown site of metastasis were excluded. For patients diagnosed with synchronous mCRC at multiple moments in time, only the first diagnosed CRC was included in the current analysis. In case of synchronous multiple CRC, the tumor with the most aggressive tumor characteristics was used.

Patients were stratified by primary tumor location: right colon (proximal to the splenic flexure), left colon (splenic flexure to rectum), and rectum. Patients’ vital status was obtained by linking the NCR to the Municipal Personal Records Database. Follow-up was completed until January 1, 2018.

### Statistical Analyses

Patient and tumor characteristics were analyzed per primary tumor location. Because cN is not a reliable parameter for lymph node stage, only (y)pT- and (y)pN-stage were presented for patients who underwent surgery.^13^ Differences in dichotomous outcomes were assessed using the *χ*^2^ test. The sites of metastases per primary tumor location were analyzed both for single sites of metastases as well as for overall metastatic sites.

Age standardized relative survival was calculated for all patients stratified to primary tumor location, as the ratio of the survival observed among the CRC patients and the survival that would have been expected based on age, gender, and year of the corresponding general population (Pohar Perme method). Using relative survival, multivariable relative excess risks (RER) were estimated with 95% confidence intervals (CI) to determine the association between risk of death and primary tumor location. Second, relative survival was calculated for patients with a single site of metastasis (liver, peritoneum, lung, or extraregional lymph nodes), stratified to primary tumor location, and RERs were estimated per single site of metastasis.

A multivariable logistic regression analysis was applied to determine the association between undergoing a metastasectomy and primary tumor location, for patients with the liver-only metastases. RERs were estimated for patients with liver-only metastases, stratified to patients that underwent metastasectomy or not.

*p* values < 0.05 were considered statistically significant. Analyses were performed in STATA (version 13.0, Statcorp LP, College Station, TX) and SPSS Statistics for Windows (version 22.0).

## Results

Between 1989 and 2014, a total of 36,297 patients were diagnosed with synchronous mCRC in the Netherlands (Table 1). There were 12,889 patients with RCC, 14,355 with LCC, and 9053 with rectal cancer. The majority of rectal cancer patients was male (62%), compared with 52% in RCC and 38% in LCC. Mucinous and signet-ring adenocarcinoma were most common in RCC, with 16% and 3% respectively versus 10% and 1% in LCC, and 6% and 1% in rectal cancer.

**Table 1:** Patient/tumor characteristics for synchronous mCRC, stratified to primary tumor location

	Primary tumor location			Total (*N* = 36,297)	*p* value (*χ*^2^)
	Right colon (*N* = 12,889)	Left colon (*N* = 14,355)	Rectum (*N* = 9053)		
Gender					
Male	6252 (49)	8311 (58)	5612 (62)	20,175 (56)	
Female	6637 (51)	6044 (42)	3441 (38)	16,122 (44)	< 0.001
Age-category					
< 45	352 (3)	509 (4)	371 (4)	1232 (3)	
45–59	2215 (17)	2954 (21)	2152 (24)	7321 (20)	
60–74	5795 (45)	6667 (46)	4129 (46)	16,591 (46)	
≥ 75	4527 (35)	4225 (29)	2401 (27)	11,153 (31)	< 0.001
Histology					
Adenocarcinoma	10,364 (80)	12,748 (89)	8368 (92)	31,480 (87)	
Mucinous adenocarcinoma	2114 (16)	1398 (10)	586 (6)	4098 (11)	
Signet ring cell carcinoma	411 (3)	209 (1)	99 (1)	719 (2)	< 0.001
T-stage					
(y)pT0-2	277 (4)	409 (5)	411 (12)	1097 (6)	
(y)pT3-4	7233 (94)	7986 (94)	2.791 (84)	18,010 (92)	
(y)pT unknown	148 (2)	107 (1)	115 (3)	370 (2)	< 0.001
N-stage					
(y)pN0	1272 (17)	1806 (21)	867 (25)	3945 (20)	
(y)pN1-2	5861 (76)	6104 (72)	2293 (67)	14,258 (73)	
(y)pN unknown	529 (7)	608 (7)	267 (8)	1404 (7)	< 0.001
Number of metastatic sites					
1	8517 (66)	9979 (70)	6293 (70)	24,789 (68)	< 0.001
≥ 2	4372 (34)	4376 (30)	2760 (30)	11,508 (32)	
Treatment					
Resection primary tumor	7686 (60)	8524 (59)	3443 (38)	19,653 (54)	< 0.001
Chemotherapy	6375 (49)	7589 (53)	4930 (54)	18,894 (52)	< 0.001
Radiotherapy	81 (1)	376 (3)	3719 (41)	4176 (12)	< 0.001
Metastasectomy	1398 (11)	1863 (13)	1434 (16)	4695 (13)	< 0.001

### Locations of Synchronous Metastases

**Fig. 1:**
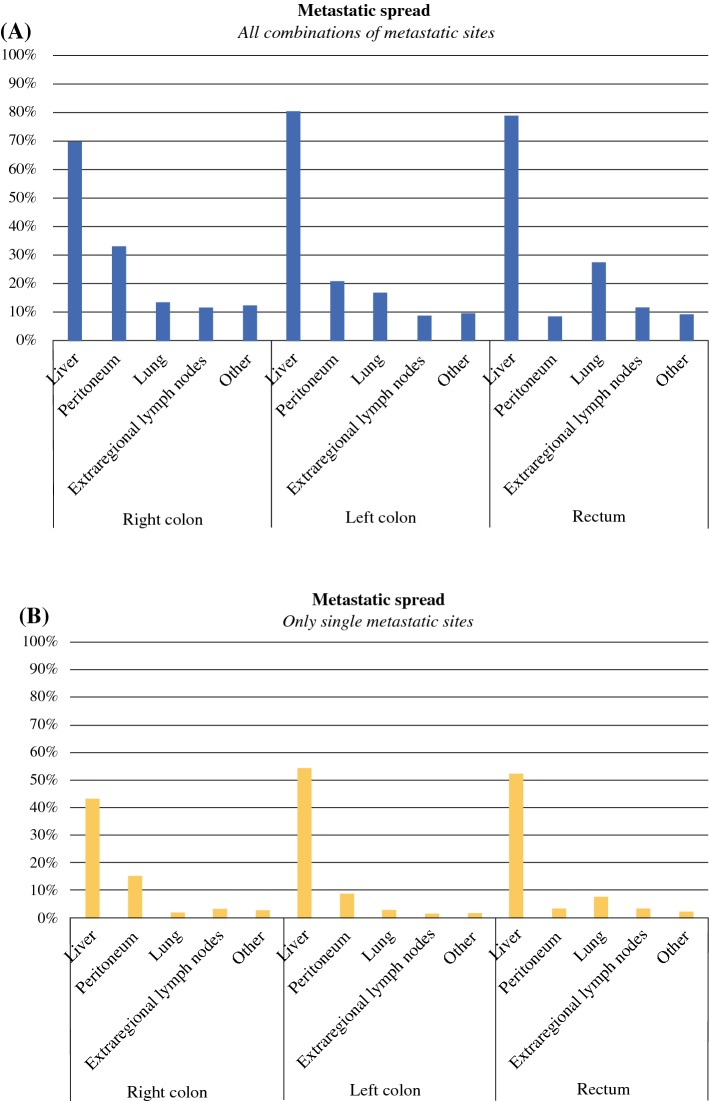
Metastatic spread to different organs according to primary tumor location. \**a** Overall prevalence of metastatic sites. **b** Prevalence of single metastatic sites (i.e., no metastasis detected elsewhere). The differences in frequencies of metastatic sites between primary tumor sites were statistically significant, *p* < 0.001) in the *χ*^2^ test

Metastatic sites differed between primary tumor locations (Fig. 1). Lung metastases were most common in rectal cancer patients (28% vs. 14% in RCC patients (*p* < 0.001) and 17% in LCC patients (*p* < 0.001)). Liver-only metastases were found in 43%, 54%, and 52% for RCC, LCC, and rectal cancer patients, respectively (*p* < 0.001). The peritoneum was the solitary metastatic site in 15%, 9%, or 4% of patients with RCC, LCC, or rectal cancer respectively (*p* < 0.001).

### Relative Survival, Including All Patients with Synchronous mCRC

**Fig. 2:**
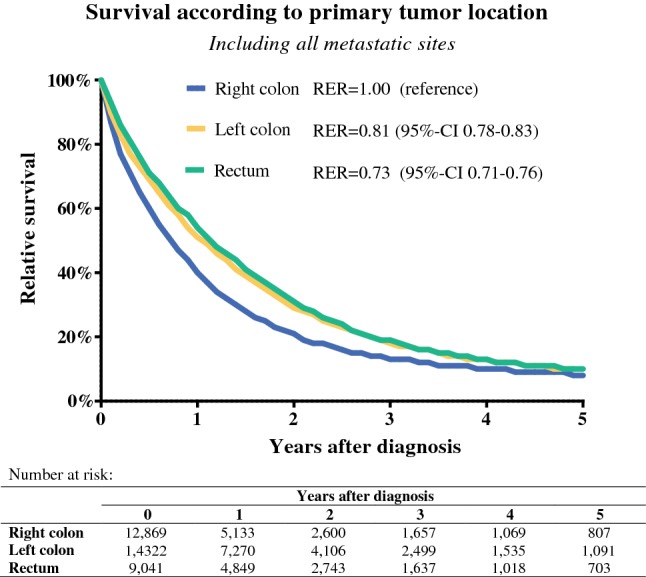
Relative survival including all metastatic sites, stratified to primary tumor location. Multivariable excess risks (RER) for death after diagnosis of synchronous mCRC are also presented (variables included in the model were location primary tumor, gender, age, morphology, period of diagnosis, resection of the primary tumor, chemotherapy, metastasectomy, and radiotherapy)

RCC had worse 1-year relative survival of 40% compared with 51% for LCC and 54% for rectal cancer (Fig. 2). RERs for death also are shown after correction for location of the primary tumor, gender, age, morphology, period of diagnosis, resection of the primary tumor, chemotherapy, metastasectomy, and radiotherapy. The relative survival was significantly worse for RCC compared with LCC (RER 0.81, 95% CI 0.78–0.83) and rectal cancer (RER 0.73, 95% CI 0.71–0.76).

### Relative Survival per Solitary Metastatic Site

**Fig. 3:**
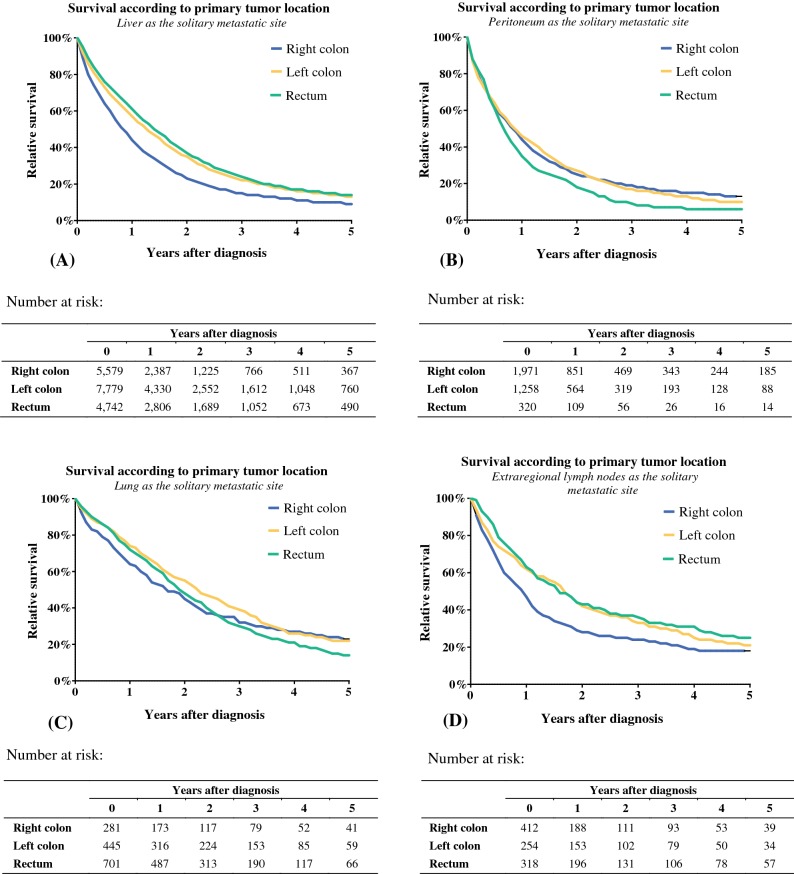
Relative survival for patients with a solitary metastatic site. Survival was analyzed per metastatic site and stratified to primary tumor location. **a** Patients with liver-only metastases. **b** Patients with the peritoneum as solitary metastatic site. **c** Patients with the lung solitary metastatic site. **d** Patients with extraregional lymph nodes as solitary metastatic sites

To exclude the possible biases of multiple metastatic sites, patients with solitary metastatic sites were analyzed for survival. The results are depicted in Fig. 3. For patients with liver-only metastases, 1-year relative survival was 43%, 57%, and 60% for RCC, LCC, and rectal cancer respectively. RCC patients had a significantly worse relative survival compared with LCC (RER 0.79, 95% CI 0.76–0.82) and rectal cancer patients (RER 0.74, 95% CI 0.70–0.77; Table 2).

**Table 2:** Multivariable relative excess risks for death after diagnosis of synchronous mCRC. Analyses were performed for patients with liver-only, lung-only, peritoneum-only, or extraregional lymph nodes-only metastases, and stratified to metastatic site

Characteristic	Liver				Peritoneum				Lung				Extraregional lymph nodes			
	*N*	RER	95% CI		*N*	RER	95% CI		*N*	RER	95% CI		*N*	RER	95% CI	
			Lower	Upper			Lower	Upper			Lower	Upper			Lower	Upper
Location primary tumor																
Right colon	5579	1.00	(Reference)		1971	1.00	(Reference)		281	1.00	(Reference)		412	1.00	(Reference)	
Left colon	7779	0.79	0.76	0.82	1258	0.88	0.72	1.06	445	0.96	0.88	1.04	254	0.78	0.65	0.94
Rectum	4742	0.74	0.70	0.77	320	0.92	0.75	1.13	701	0.98	0.84	1.14	318	0.70	0.55	0.88
Gender																
Male	10,659	1.00	(Reference)		1770	1.00	(Reference)		791	1.00	(Reference)		489	1.00	(Reference)	
Female	7441	1.07	1.03	1.10	1779	1.02	0.89	1.16	636	0.97	0.90	1.05	495	1.11	0.95	1.29
Age at diagnosis (yearr)																
< 45	545	1.00	(Reference)		153	1.00	(Reference)		29	1.00	(Reference)		67	1.00	(Reference)	
45–59	3745	0.94	0.85	1.03	588	1.43	0.89	2.30	184	1.27	1.03	1.56	203	0.93	0.66	1.29
60–74	8415	0.99	0.90	1.09	1464	1.47	0.93	2.32	598	1.30	1.07	1.59	403	1.17	0.86	1.61
75+	5395	1.10	1.00	1.22	1344	1.48	0.92	2.37	616	1.61	1.31	1.97	311	1.26	0.90	1.78
Morphology																
Adenocarcinoma	16,667	1.00	(Reference)		2172	1.00	(Reference)		1289	1.00	(Reference)		835	1.00	(Reference)	
Mucinous adenocarcinoma	1370	1.07	1.00	1.14	1065	1.14	0.90	1.44	129	0.87	0.80	0.95	104	0.95	0.74	1.21
Signet Ring Cell carcinoma	63	1.37	1.05	1.79	312	1.63	0.76	3.53	9	1.48	1.30	1.68	45	1.74	1.24	2.44

Variables also included in the model were the period of diagnosis, resection of the primary tumor, chemotherapy, metastasectomy, and radiotherapy

RCC patients with extraregional lymph nodes as the single site of metastasis had a worse relative survival compared with LCC (RER 0.78, 95% CI 0.65–0.94) and rectal cancer patients (RER 0.70, 95% CI 0.55–0.88). Differences in survival for patients with peritoneal or pulmonary metastases were less pronounced, with RERs that did not differ significantly.

### Patients with Liver-Only Metastases

Patients with RCC had a lower chance of undergoing a metastasectomy compared with patients with LCC (OR 1.27, 95% CI 1.13–1.43) or rectal cancer (OR 1.20, 95% CI 0.99–1.45; Table 3). In Table 4, RERs are presented for patients with liver-only metastases, stratified for metastasectomy status. In both groups, patients with RCC showed worse survival compared with LCC.

**Table 3:** Logistic regression for the chance of undergoing a metastasectomy in patients with liver-only metastases

Characteristic	Liver-only metastases			
	*N*	OR	(95% CI)	
			Lower	Upper
Location primary tumor				
Right colon	5586	1.00	(Reference)	
Left colon	7802	1.27	1.13	1.43
Rectum	4747	1.20	0.99	1.45
Gender				
Male	10,678	1.00	(Reference)	
Female	7457	0.95	0.86	1.05
Age (yr)				
< 45	546	1.00	(Reference)	
45–59	3748	0.73	0.56	0.95
60–74	8428	0.53	0.41	0.69
≥ 75	5413	0.20	0.15	0.27
Morphology				
Adenocarcinoma	16,698	1.00	(Reference)	
Mucinous adenocarcinoma	1374	0.90	0.75	1.08
Signet Ring Cell Carcinoma	63	0.77	0.31	1.94

Variables also included in the model were the period of diagnosis, resection of the primary tumor, chemotherapy, metastasectomy, and radiotherapy

**Table 4:** Multivariable relative excess risks for death after diagnosis of synchronous mCRC. Patients with liver-only metastases were selected and stratified to undergoing metastasectomy or not

Characteristic	No metastasectomy				Metastasectomy			
	*N*	RER	95% CI		*N*	RER	95% CI	
			Lower	Upper			Lower	Upper
Location primary tumor								
Right colon	4901	1.00	(Reference)		678	1.00	(Reference)	
Left colon	6586	0.80	0.77	0.84	1193	0.66	0.57	0.76
Rectum	3823	0.73	0.70	0.77	919	0.84	0.66	1.06
Gender								
Male	8921	1.00	(Reference)		1738	1.00	(Reference)	
Female	6389	1.06	1.02	1.10	1052	1.15	1.02	1.29
Age (yr)								
< 45	405	1.00	(Reference)		140	1.00	(Reference)	
45–59	2958	0.95	0.85	1.05	787	0.90	0.70	1.16
60–74	6936	1.01	0.91	1.13	1479	0.87	0.68	1.11
75 +	5011	1.10	0.99	1.22	384	0.96	0.71	1.29
Morphology								
Adenocarcinoma	14,079	1.00	(Reference)		2588	1.00	(Reference)	
Mucinous adenocarcinoma	1174	1.07	1.00	1.14	196	1.07	0.86	1.34
Signet ring cell carcinoma	57	1.36	1.03	1.79	6	1.52	0.54	4.27

Variables also included in the model were period of diagnosis, resection of the primary tumor, chemotherapy, metastasectomy, and radiotherapy

## Discussion

In this large population-based study, primary tumor location was identified as an independent prognostic factor for relative survival. Also, the frequency of different metastatic sites varied based on primary tumor location. These results support the notion that RCC, LCC, and rectal cancer should be regarded as separate entities.

Patients with RCC had a significantly worse 1-year relative survival of 40% compared with more than 50% for patients with LCC and rectal cancer. In patients with liver-only metastases, the relative survival remained significantly worse for RCC compared with LCC and rectal cancer. These differences in survival are in line with previous reports, suggesting a prognostic impact of primary tumor location in mCRC.^4^,^10^,^14^,^15^ However, timing of metastatic disease, by the distinction of metachronous or synchronous mCRC, is a prognostic factor as well.^16^ Unfortunately, most previous studies made no clear distinction between synchronous and metachronous metastases in their analyses.^4^,^8^,^15^ Therefore, the current study adds new information on the significance of primary tumor location in synchronous mCRC patients.

The explanation for the differences in survival according to the primary tumor locations is most likely multifactorial. A part of this explanation is the difference in metastatic sites, as observed in relation to primary tumor location. These findings are consistent with previous epidemiologic research that indicates a relationship between primary tumor location and metastatic location.^17^–^19^ The results presented in the current paper add valuable information, because previous publications are limited by their relatively small sample size, grouping together of metachronous and synchronous metastases, or the absence of several sites of metastasis.^19^ The differences in metastatic sites can be explained by several hypotheses.^17^,^18^,^20^ First, there is the seed-and-soil hypothesis, which states that tumor metastases have a preference for specific organs (e.g., the liver), based on interactions between tumor cells and their microenvironment.^21^ New insights, such as metastatic gene signatures and tumor-stroma interactions at a molecular level, have further refined this hypothesis.^22^ Previously, data from an autopsy study, including more than 1500 patients with metastatic CRC demonstrated significant differences in metastatic sites between histological subtypes, with a higher rate of peritoneal metastasis as well as multiple sites of metastasis for both mucinous and signet ring cell adenocarcinoma.^9^ In the current study, we observed a higher prevalence of mucinous and signet ring cell adenocarcinoma for RCC, yielding a possible explanation for the higher rate of peritoneal metastases in RCC. Second, distinct vascular drainage systems explain, according to the hemodynamic hypothesis, different metastatic sites. This is most evident for the increased numbers of lung metastases derived from the rectum, because venous drainage of the rectum bypasses the portal system and encounters the central circulation first.^2^,^18^,^19^ The differences in metastatic sites could be partly responsible for differences in survival between primary tumor locations. Peritoneal metastases, which are associated with poor survival, were more commonly found for RCC.^23^ Liver and pulmonary metastases, which can potentially be treated with curative intent, were more prevalent in LCC and rectal cancer.

However, the difference in metastatic sites is not the only factor that explains the prognostic difference observed between RCC, LCC, and rectal cancer, because survival differences persisted when patients with liver-only metastases were selected. In this group, RCC patients less frequently underwent surgical resection, maybe because of a higher tumor load. It has been suggested that the relative delay in diagnosis of RCC is at least partly responsible for this.^14^,^24^,^25^

Still, the difference in survival remained present even when only patients with metastasectomy were selected. This suggests that underlying biology of RCC differs from LCC and rectal cancer, with increased numbers of cases with BRAF mutations and KRAS mutations.^8^,^15^,^26^

Data in this study were derived from the NCR which is known for its high-quality data and complete registration. We aimed to minimize selection bias by selecting all patients with synchronous mCRC in the Netherlands, hereby representing daily practice. There are, however, several limitations that should be addressed. The major limitation of this retrospective study was, as in most population-based studies, the absence of information on certain patient and tumor characteristics. First, the NCR did not register data on comorbidity, performance status, and details of treatment regimens during our study period, which may have differed between primary tumor sites, thereby influencing survival outcomes. Second, and possibly most importantly, data on molecular profiling was lacking while previous studies showed significant differences in molecular profiles between primary tumor sites. Analyses of mutational profiles even suggest that the current right/left classification may not fully recapitulate regional variations in tumor biology.^27^ Because data on mutational profiles were lacking in the current study, it is impossible to state anything to this extend.

## Conclusions

We showed significant differences in relative survival between synchronous metastasized RCC, LCC, and rectal cancer, which can be partly explained by the distinct metastatic sites, and are also likely to be caused by differences in tumor biology. These findings support the concept that primary tumor location should be regarded as a prognostic factor in synchronous mCRC and emphasizes its implication for clinical practice. Future trials on the effect of chemotherapy should be stratified to primary tumor location, because it is likely to effect the therapeutic response.
